# Characterization of the complete mitochondrial genome of *Aspergillus terricola* (Aspergillaceae, Eurotiales), isolated from soy sauce fermentation system

**DOI:** 10.1080/23802359.2021.2008832

**Published:** 2021-12-22

**Authors:** Yue Deng, Jie He

**Affiliations:** School of China Alcoholic Drinks, Luzhou Vocational and Technical College, Sichuan, Luzhou, P. R. China

**Keywords:** *Aspergillus*, mitochondrial genome, phylogenetic analysis

## Abstract

In the present study, the complete mitochondrial genome of *Aspergillus terricola* É.J. Marchal 1893 was sequenced and assembled. The mitochondrial genome of *A. terricola* was composed of circular DNA molecules, with a total size of 28,689 bp. The GC content of the *A. terricola* mitochondrial genome was 26.34%. A total of 18 protein-coding genes (PCGs), 2 ribosomal RNA (rRNA) genes, and 26 transfer RNA (tRNA) genes were detected in the *A. terricola* mitochondrial genome. Phylogenetic analysis based on the combined mitochondrial gene dataset indicated that the *A. terricola* exhibited a close relationship with *A. parasiticus*.

*Aspergillus* is a highly diverse genus, widely distributed in environments, including soil, water, plant tissue and so on. Up to now, hundreds of species have been described in the genus *Aspergillus*. More and more attention has been paid to the research of *Aspergillus*, which plays an important role in agriculture, industry and food research (Facchini et al. 2012; Michelin et al. 2012). *Aspergillus terricola* É.J. Marchal 1893 has been isolated from fermented products (Facchini et al. 2012). It can produce high activity protease, including acidic, neutral and alkaline protease, and has been paid more and more attention in the food industry (Yadav et al. 2009; Michelin et al. 2010; Singh et al. 2010; Michelin et al. 2011). The mitochondrial gene is an important molecular marker to analyze the phylogenetic relationship and evolution of species (Zhang et al. 2017; Wu et al. 2021; Q Li, L Li, et al. 2021). However, up to now, the mitochondrial genome characteristics of *A. terricola* have not been understood. This study served as the first report on the complete mitochondrial genome of *A. terricola*, which will promote the understanding of phylogeny and evolution of this important fungal species.

The specimen (*A. terricola*) was collected from Luzhou, Sichuan, China (105.40 E; 28.91 N) from the soy sauce fermentation system. A specimen was deposited in the collection center of Luzhou Vocational and Technical College (Yue Deng, 157317724@qq.com) under the voucher number L03. The complete mitochondrial genome of *A. terricola* was sequenced and assembled according to previously described methods (Li et al. 2019; Wang, Song, et al. 2020; X Wang, YJ Wang, et al. 2020). In brief, genomic DNA of *A. terricola* was extracted using a fungal DNA kit (Cat. #D3390-00, Omega Bio-Tek, Norcross, GA, USA) according to the manufacturer’s instructions. NEBNext Ultra II DNA Library Prep Kits (NEB, Beijing, China) were used to construct sequencing libraries. Whole genomic sequencing was performed by the Illumina HiSeq 2500 Platform (Illumina, San Diego, CA, USA). We conducted quality control steps to obtain clean reads from the raw sequencing reads according to previous studies (Li, Yang, et al. 2020). The complete mitochondrial genome of *A. terricola* was assembled using NOVOPlasty v4.3.1 with the obtained clean reads under the k-mer size of 27 (Dierckxsens et al. 2017; Li, Ren, et al. 2020). The complete mitochondrial genome of *A. terricola* was annotated using MITOS (Bernt et al. 2013) and MFannot (Valach et al. 2014) based on the genetic code 4. PCGs or ORFs in the *A. terricola* mitochondrial genome were predicted using the NCBI Open Reading Frame (ORF) Finder (NCBI Resource Coordinators 2018) and annotated by BLASTP searches against the NCBI non-redundant protein sequence database (Bleasby and Wootton 1990). We then predicted the tRNA genes in the *A. terricola* mitochondrial genome using tRNAscan-SE v1.3.1 (Lowe and Chan 2016).

The complete mitochondrial genome of *A. terricola* is 28,689 bp in length. The base composition of the *A. terricola* mitochondrial genome is as follows: A (35.82%), T (37.83%), G (14.55%) and C (11.80%). A total of 18 PCGs (*cox1*, *cox2*, *cox3*, *cob*, *atp6*, *atp8*, *atp9*, *nad1*, *nad2*, *nad3*, *nad4*, *nad4L*, *nad5*, *nad6*, *rps3*, *orf205*, *orf153*, and *orf650*), 2 ribosomal RNA genes (*rns* and *rnl*), and 26 transfer RNA genes were detected in the *A. terricola* mitochondrial genome. The mitochondrial genome of *A. terricola* contained 3 introns, 2 of which were located in *cox1*and one was located in the *rnl* gene. Two introns belong to an unknown type, and the other belongs to group I. We named these introns according to the previously described method (S Zhang and YJ Zhang 2019). To reveal the phylogenetic relationships of the Eurotiales species, a phylogenetic tree for 17 Eurotiales species was constructed. Single mitochondrial genes were first aligned by using MAFFT v7.037 (Katoh et al. 2019), and then concatenated into a pseudogene dataset by using SequenceMatrix v1.7.8 (Vaidya et al. 2011). We detected best-fit models of evolution and partitioning schemes using PartitionFinder 2.1.1 (Lanfear et al. 2017). The phylogenetic relationships of the 17 Eurotiales species were analyzed by using RAxML v 8.0.0 (Stamatakis 2014). The maximum likelihood method (ML) method was used to construct the phylogenetic tree based on the combined 14 core PCGs (Cheng et al. 2021; Li, Wu, et al. 2021). We assessed bootstrap values (BS) through an ultrafast bootstrap approach, with 10,00 replicates. As shown in the phylogenetic tree (Figure 1), the mitochondrial genome of *A. terricola* exhibited a close relationship with *A. parasiticus*.

**Figure 1.**
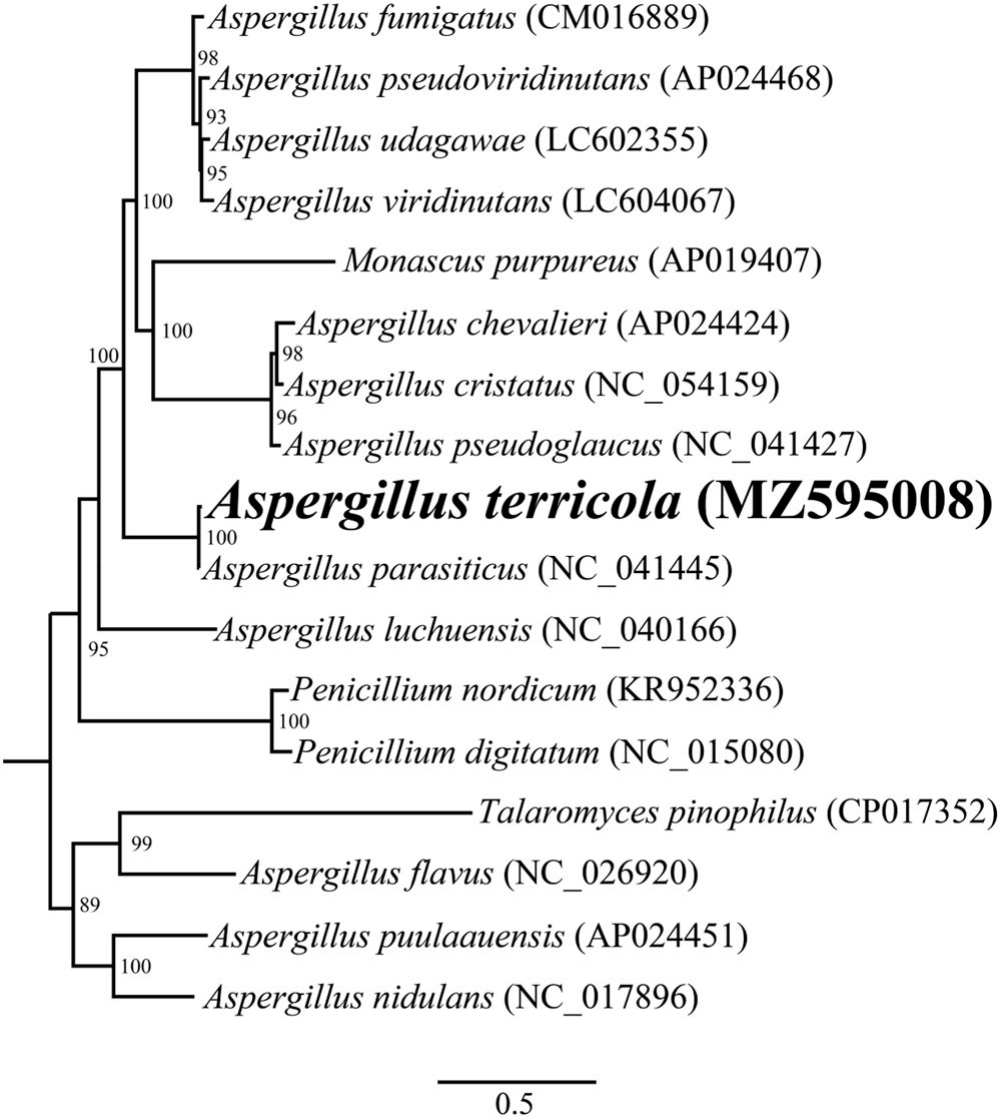
Phylogenetic analysis of 17 Eurotiales species using maximum likelihood method based on the combined 14 core protein-coding genes. Accession numbers of mitochondrial sequences used in the phylogenetic analysis are listed in brackets after species.

## Data Availability

The genome sequence data that support the findings of this study are openly available in GenBank of NCBI at (https://www.ncbi.nlm.nih.gov/) under the accession no. MZ595008. The associated BioProject, SRA, and Bio-Sample numbers are PRJNA747851, SRR15184095, and SAMN20297433, respectively.
